# Efficacy and tolerability of vortioxetine monotherapy in SSRI-resistant OCD: a retrospective multicenter study

**DOI:** 10.3389/fpsyt.2025.1617345

**Published:** 2025-06-13

**Authors:** Vassilis Martiadis, Enrico Pessina, Carlo Ignazio Cattaneo, Azzurra Martini, Fabiola Raffone, Tiziano Prodi, Miriam Olivola, Domenico De Berardis, Beatrice Benatti, Bernardo Maria Dell’Osso

**Affiliations:** ^1^ Department of Mental Health, Asl Napoli 1 Centro, Naples, Italy; ^2^ Department of Mental Health, Asl Cuneo 2, Bra, Italy; ^3^ Department of Mental Health, Asl Biella, Biella, Italy; ^4^ Department of Biomedical and Clinical Sciences Luigi Sacco, Department of Psychiatry, ASST Fatebenefratelli-Sacco, University of Milan, Milan, Italy; ^5^ Department of Mental Health, Asl Teramo, Teramo, Italy; ^6^ Department of Psychiatry and Behavioral Sciences, Bipolar Disorders Clinic, Stanford Medical School, Stanford University, Stanford, CA, United States; ^7^ CRC ‘Aldo Ravelli’ for Neurotechnology & Experimental Brain Therapeutics, Milan, Italy; ^8^ Centro per lo Studio dei Meccanismi Molecolari alla base delle Patologie neuro-psico-geriatriche, University of Milan, Milan, Italy

**Keywords:** vortioxetine, obsessive-compulsive disorder, treatment-resistant, efficacy, tolerability, SSRI-resistant

## Abstract

**Introduction:**

Treatment-resistant obsessive-compulsive disorder (OCD) remains a major clinical challenge, with a substantial proportion of patients failing to respond to standard treatment with selective serotonin reuptake inhibitors (SSRI). Vortioxetine, a multimodal antidepressant approved for major depressive disorder, has shown potential advantages in terms of tolerability and cognitive enhancement, but its efficacy in OCD has not been systematically explored.

**Methods:**

This multicenter, retrospective, observational study analyzed the clinical records of 64 adult patients with a DSM-5 diagnosis of OCD who had failed to respond to at least one adequate SSRI trial and were treated with vortioxetine monotherapy (minimum dose: 20 mg/day; duration: ≥8 weeks). The primary outcome was reduction in total Y-BOCS score. Secondary outcomes included changes in HAM-D and HAM-A scores and frequency of adverse events.

**Results:**

At week 8, 39.1% of patients met responder criteria (≥25% reduction in total Y-BOCS score). The mean Y-BOCS score decreased from 27.1 to 20.7 (p < 0.001). HAM-D and HAM-A scores showed significant improvements (HAM-D: from 21.0 to 12.6; HAM-A: from 26.9 to 16.1; both p < 0.001). The treatment was well tolerated, with nausea (29.7%) and sedation (18.8%) being the most common side effects; no serious adverse events occurred.

**Conclusion:**

This study provides preliminary evidence of the efficacy and tolerability of vortioxetine monotherapy in SSRI-resistant OCD. The observed improvements in OCD, depressive and anxiety symptoms suggest that vortioxetine may represent a valuable therapeutic option. Further prospective controlled trials are needed to confirm these findings.

## 1 Introduction

Obsessive-compulsive disorder (OCD) is a chronic and distressing psychiatric disorder characterized by both intrusive thoughts (obsessions) and repetitive behaviors or mental actions (compulsions) (1). The global prevalence of the disorder is estimated at 2-3%, with a profound impact on individual functioning, quality of life and healthcare costs (2). Standard first-line treatments include selective serotonin reuptake inhibitors (SSRIs) and cognitive behavioral therapy, often with exposure and response prevention (3). However, despite these well-established interventions, a significant proportion of patients show only a partial response, requiring alternative pharmacological approaches to achieve adequate symptom control (4).

Furthermore, both pharmacological and placebo interventions are generally less effective in treating OCD than other anxiety disorders (5). Meta-analytic evidence suggests that the placebo effect in OCD trials is significantly lower than in generalized anxiety disorder, panic disorder and post-traumatic stress disorder, suggesting a higher threshold for symptom relief and a need for novel therapeutic strategies (5, 6). Treatments such as SSRIs, which are the most commonly used first-line pharmacological approach, usually show moderate responses, with approximately 40-60% of patients reporting residual symptoms and up to 30% considered treatment-resistant (4, 7). Current augmentation strategies, such as the addition of atypical antipsychotics, have shown some efficacy, but their side effects burden often limits long-term use (2, 8, 9). Among second generation antipsychotics risperidone and aripiprazole exhibit more robust evidences (10, 11), while paliperidone, quetiapine and olanzapine showed inconsistent results failing to outperform placebo in meta-analyses (12, 13). Recently, both cariprazine and brexpiprazole have shown preliminary promising safety and efficacy among resistant OCD augmentation strategies (14–16) but these results need confirmation on larger populations. Given these limitations, there is growing interest in vortioxetine as a potential monotherapy for treatment resistant OCD.

Vortioxetine, a multimodal serotonergic agent, is primarily approved for major depressive disorder (MDD), but its unique pharmacodynamic properties have attracted interest as a potential treatment for OCD (17, 18). Unlike conventional SSRIs, vortioxetine acts as a serotonin transporter inhibitor while modulating multiple serotonin receptors, including 5-HT1A agonism and 5-HT3, 5-HT7 and 5-HT1D antagonism. This pharmacological profile may improve cognitive flexibility and emotional regulation, both implicated in OCD (19). In addition, the pharmacokinetic profile of vortioxetine, characterized by a half-life of approximately 66 hours and minimal interactions with cytochrome P450 enzymes, may offer advantages in tolerability and dosing convenience compared to other serotonergic antidepressants (18).

Moreover, preliminary studies suggest that its multimodal action may provide benefits beyond those of conventional SSRIs, particularly in addressing cognitive rigidity, which is a core feature of OCD (20). In addition, its favorable tolerability profile and lower propensity for sexual dysfunction or weight gain compared with other antidepressant agents may improve adherence, overall treatment outcomes and satisfaction (18).

Despite its theoretical advantages, clinical data on the efficacy of vortioxetine in OCD remain scarce. To date, no study has systematically evaluated its impact on OCD symptomatology. This study aims to fill this gap by presenting real-world data on the efficacy and tolerability of vortioxetine monotherapy in a sample of 64 patients diagnosed with OCD who have failed to respond to SSRI treatment.

## 2 Subjects and methods

This is a multicenter, retrospective, observational study analyzing the clinical records of inpatients and outpatients diagnosed with OCD according to DSM-5 criteria (1). Patients were treated between January 2023 and December 2024 at the Mental Health Departments of Alba-Bra, Biella, Naples, and Teramo, as well as at the Department of Biomedical and Clinical Sciences Luigi Sacco, University of Milan, Italy. To be included in the analysis patients had to meet the following criteria: age ≥18 years; a total score of ≥16 on the Yale-Brown Obsessive-Compulsive Scale (Y-BOCS) (21); documented failure to respond to at least one adequate trial of a SSRI, defined as insufficient response after a minimum of 12 weeks at a therapeutic dosage; completion of at least 8 weeks of treatment with vortioxetine monotherapy; availability of complete sociodemographic and clinical data, including the Y-BOCS, the Hamilton Depression Rating Scale (HAM-D) (22), and the Hamilton Anxiety Rating Scale (HAM-A) (23) scores at baseline, week 2, week 4, week 6 and week 8. Insufficient response was defined as less than 25% reduction in Y-BOCS score, based on clinical assessments. Although some guidelines recommend defining treatment resistance as failure of two or more SSRI trials, we adopted a more inclusive criterion to reflect real-world prescribing practices and maximize generalizability. The most frequently used SSRIs before vortioxetine initiation were sertraline (100–200 mg/day), fluoxetine (40–60 mg/day), and escitalopram (15–20 mg/day). All were administered at therapeutic dosages for a minimum of 12 weeks. Due to the retrospective design, individual medication histories were not fully available for all patients. All patients received vortioxetine at a minimum target dose of 20 mg/day, which was reached within the first 7–14 days of treatment. Initial dosing and titration were determined by clinical judgement. The use of other psychotropic medications, such as SSRIs, tricyclic antidepressants and antipsychotics, was not allowed. Concomitant medications used prior to vortioxetine initiation (e.g., for sleep or anxiety management) were allowed and recorded, but no additional psychotropic drugs were introduced during the 8-week observation period. This study was conducted in accordance with the principles of the Declaration of Helsinki. Ethical approval was not required, as this was a retrospective analysis of anonymized clinical data collected during routine care. All patients provided written informed consent for the use of their anonymized clinical data for research and educational purposes. Specific informed consent was also obtained for the off-label use of vortioxetine, which is approved in Italy solely for the treatment of MDD (maximum licensed dose: 20 mg/day). Sociodemographic, clinical, and safety data were extracted from medical records. Patients were assessed in accordance with routine clinical practice at baseline and at weeks 2, 4, 6, and 8 of treatment. All diagnoses and assessments were carried out by psychiatrists with extensive clinical experience. Treatment response was defined as a reduction of ≥25% in the total Y-BOCS score from baseline to week 8. Depressive and anxiety symptoms were assessed using the HAM-D and the HAM-A. All adverse events, whether spontaneously reported by the patients or observed by clinicians, were documented using the UKU Side Effect Rating Scale (24). Statistical analyses were conducted using IBM SPSS Statistics Software, version 19. Paired t-tests were used to assess differences in Y-BOCS, HAM-D, and HAM-A scores between baseline and week 8. Repeated measures ANOVA was also performed to evaluate changes in these scores over time. Statistical significance was set at p-value < 0.05.

## 3 Results

A total of 64 patients met inclusion criteria and were included in the analysis. **Tables 1**, **2**, **3** report the sociodemographic and clinical characteristics of the sample. The mean daily dose of vortioxetine was 25.9 mg (± 6.2); 6 patients (9.4%) received the dose of 40 mg/day.

**Table 1:** Socio-demographic and clinical characteristics of the sample.

Parameters	Value (n, % or mean ± SD)	
Age, years (mean ± SD)		40.3 ± 10.7
Sex, n (%)		
	Male	42 (65.6)
	Female	22 (34.4)
Marital status, n (%)		
	Single	25 (39.1)
	Married	27 (42.1)
	Divorced	8 (12.5)
	Widowed	4 (6.3)
Educational level, years (mean ± SD)		13.2 ± 3.5
Working for pay, n (%)		
	Yes	33 (51.6)
	No	31 (48.4)
Age at onset, years (mean ± SD)		20.4 ± 5.0

**Table 2:** Obsessive-compulsive symptomatology (according to YBOCS Checklist).

Symptom type	n (%)
Obsessions, n (%)	
Aggressive	38 (59.4)
Contamination	21 (32.8)
Need for symmetry	18 (28.1)
Religious	6 (9.4)
Somatic	6 (9.4)
Miscellaneous	4 (6.2)
Compulsions, n (%)	
Checking	36 (56.2)
Cleaning	24 (37.5)
Miscellaneous	15 (23.4)
Ordering	13 (20.3)
Repeating	12 (18.8)

**Table 3:** Psychiatric comorbidities in the sample.

Comorbidity type	n (%)
Psychiatric comorbidity, n (%)	
Yes	38 (59.4)
No	26 (40.6)
>1 disorder	6 (9.4)
Type of psychiatric comorbidity, n (%)	
Major Depression	13 (20.3)
Substance Use Disorder^1^	12 (18.8)
Generalized Anxiety Disorder	6 (9.4)
Panic Disorder	4 (6.2)
Social Phobia	3 (4.7)
Bulimia Nervosa	2 (3.1)
ADHD	1 (1.6)
Trichotillomania	1 (1.6)

^1^alcohol (n=6); THC (n=5); benzodiazepine (n=2); cocaine (n=2); gambling (n=1).

ADHD, Attention deficit hyperactivity disorder.

### 3.1 Efficacy

**Figure 1:**
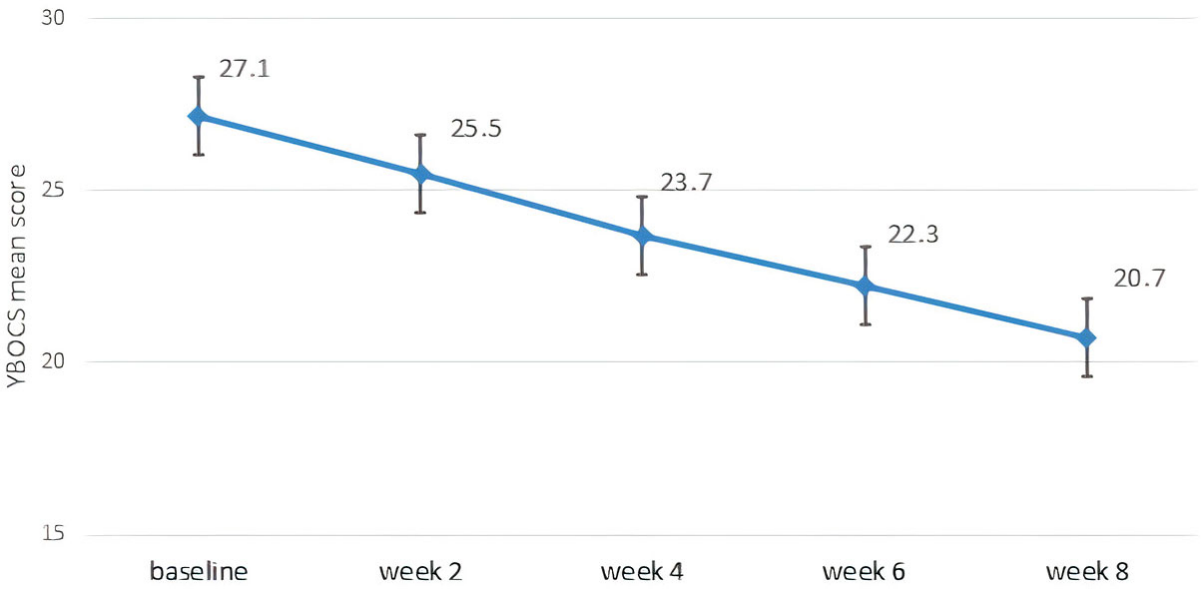
Mean modification in Y-BOCS total score across study timepoints.

A significant reduction in obsessive-compulsive symptoms was observed over the 8-week study period. The total Y-BOCS score decreased from a baseline mean of 27.1 (± 5.2) to 20.7 (± 4.2) at week 8 (p<0.001, paired t-test). Improvements were also observed in both the obsessions and compulsions subscales (**Table 4**). **Figure 1** illustrates the progressive reduction in total Y-BOCS scores across the study time points. The mean obsession score declined from 13.1 to 10.2, while the mean compulsion score declined from 14.1 to 10.5. Repeated measures ANOVA confirmed significant reductions over time for all Y-BOCS domains (total score: F=19.949, p<0.001). At endpoint, 25 patients (39.1%) met responder criteria, defined as a ≥25% reduction in total Y-BOCS score from baseline. Significant reductions in depressive and anxiety symptoms were also observed over time (**Table 5**). The mean HAM-D score decreased from 21.0 (± 5.9) at baseline to 12.6 (± 4.8) at week 8 (F=25.045, p<0.001), while the mean HAM-A score decreased from 26.9 (± 7.4) to 16.1 (± 7.1) (F= 32.729, p<0.001).

**Table 4:** Vortioxetine efficacy in reducing obsessive-compulsive symptoms (n=64).

Timepoint	Baseline (T0) Mean (± SD)	2 weeks (T1) Mean (± SD)	4 weeks (T2) Mean (± SD)	6 weeks (T3) Mean (± SD)	8 weeks (T4) Mean (± SD)	Statistics
YBOCS total	27.1 (5.2)	25.5 (4.5)	23.7 (4.2)	22.2 (3.7)	20.7 (4.2)	T0 vs T1 t=4.776 p<0.001 T1 vs T2: t=5.702 p<0.001 T2 vs T3: t=3.999 p<0.001 T3 vs T4: t=5.305 p<0.001 ANOVA: F=19.949 p<0.001
YBOCS *Obsession*	13.1 (2.7)	12.6 (2.0)	11.6 (1.7)	11.1 (1.7)	10.2 (2.3)	T0 vs T1 t=1.891 p=0.063 T1 vs T2: t=6.016 p<0.001 T2 vs T3: t=3.279 p=0.002 T3 vs T4: t=4.284 p<0.001 ANOVA: F=14.341 p<0.001
YBOCS *Compulsion*	14.1 (3.1)	12.8 (3.6)	11.9 (2.8)	11.2 (2.4)	10.5 (2.3)	T0 vs T1 t=5.123 p<0.001 T1 vs T2: t=3.221 p=0.002 T2 vs T3: t=2.816 p=0.006 T3 vs T4: t=3.712 p<0.001 ANOVA: F=22.723 p<0.001

YBOCS, Yale-brown Obsessive-Compulsive Scale.

**Table 5:** Vortioxetine efficacy in reducing depressive and anxious symptoms (n=64).

Timepoint	Baseline (T0) Mean (± SD)	2 weeks (T1) Mean (± SD)	4 weeks (T2) Mean (± SD)	6 weeks (T3) Mean (± SD)	8 weeks (T4) Mean (± SD)	Statistics
HAM-D	21.0 (5.9)	19.0 (5.6)	16.0 (4.8)	14.1 (5.0)	12.6 (4.8)	T0 vs T1 t=4.463 p<0.001 T1 vs T2: t=6.171 p<0.001 T2 vs T3: t=5.182 p<0.001 T3 vs T4: t=4.431 p<0.001 ANOVA: F=25.045 p<0.001
HAM-A	26.9 (7.4)	24.0 (6.6)	20.8 (7.4)	18.8 (7.5)	16.1 (7.1)	T0 vs T1 t=6.193 p<0.001 T1 vs T2: t=5.794 p<0.001 T2 vs T3: t=3.636 p=0.001 T3 vs T4: t=5.639 p<0.001 ANOVA: F=32.729 p<0.001

HAM-D, Hamilton Depression Rating Scale; HAM-A, Hamilton Anxiety Rating Scale.

### 3.2 Safety and tolerability

Overall, 38 patients (59.4%) reported at least one adverse event. All side effects were rated as mild. The most common was nausea or vomiting, reported by 19 patients (29.7%), followed by sleepiness or sedation (18.8%) and tremor (17.2%). A full list of reported adverse events is presented in **Table 6**. No safety issue or serious adverse events were reported.

**Table 6:** Adverse events (n=64).

Adverse events	n (%)
Nausea/vomiting	19 (29.7)
Sleepiness/Sedation	12 (18.8)
Tremor	11 (17.2)
Constipation	1 (1.6)
Diarrhea	1 (1.6)
Headache	1 (1.6)
No side effect	26 (40.6)

## 4 Discussion

This is, to our knowledge, the first clinical study to systematically evaluate vortioxetine monotherapy in patients with SSRI-resistant OCD. In our 8-week retrospective observational analysis, approximately 39% of patients achieved a ≥25% improvement in total Y-BOCS score. This finding supports the pharmacodynamic rationale suggesting that vortioxetine’s multimodal serotonergic action may benefit patients who have failed to respond to previous SSRI treatment (18, 25). While this response rate may appear modest, it is clinically meaningful in a treatment-resistant population where even partial improvement can represent a significant therapeutic gain. This response rate is broadly consistent with those observed in SSRI augmentation trials using atypical antipsychotics such as risperidone or aripiprazole, which typically show response rates around 35–40% in treatment-resistant OCD populations (10, 11). Other agents like quetiapine or olanzapine have demonstrated less robust or inconsistent efficacy (12, 13), and switching strategies—whether to a different SSRI or to clomipramine—may be limited by tolerability concerns or variable outcomes (24). In this context, vortioxetine may represent a viable monotherapy alternative, warranting further investigation. Furthermore, no patient experienced a significant worsening of OCD symptoms, highlighting the tolerability and overall safety of the drug in this difficult-to-treat population.

Given the lack of prior studies evaluating vortioxetine in OCD, direct comparisons are limited. However, findings from the TRUE study (26), a recent similar 8-week observational study in patients with comorbid MDD and generalized anxiety disorder (GAD) showed significant improvement in depressive symptoms, as measured by the Montgomery–Åsberg Depression Rating Scale (MADRS) and a marked reduction in HAM-A scores, with most patients moving from moderate/severe to mild anxiety over the observation period. Similarly, our study documented meaningful reductions in HAM-D and HAM-A scores, suggesting that vortioxetine’s antidepressant and anxiolytic properties may extend to patients with SSRI-resistant OCD. This is particularly relevant given that depressive and anxiety symptoms are common in OCD and may negatively impact global functioning and treatment response (27). A pharmacological agent capable of addressing both OCD and affective symptoms may thus offer a valuable treatment option in complex and comorbid clinical presentations. Although Badr et al. enrolled patients with MDD and GAD, the parallel findings of mood and anxiety relief, coupled with good tolerability, underscore a consistent therapeutic signal that merits further, larger confirmatory investigations.

In contrast to the TRUE study, which enrolled patients predominantly from the United Arab Emirates, the RECONNECT trial (28) was carried out in European and Asian countries which are often considered more similar to our local healthcare context. This study involved adult outpatients diagnosed with severe MDD with comorbid severe GAD. Notably, RECONNECT mandated a forced up-titration from 10 mg to 20 mg of vortioxetine after the first week, resulting in almost all patients reaching and maintaining the 20 mg dose for 8 weeks. This protocol highlighted both the feasibility and tolerability of early dose escalation in a difficult-to-treat group. In our study, the average dose of vortioxetine was 26 mg/day, with titration up to 40 mg/day in 10% of patients. Although only a subset of our patients received higher dosages, the results still support the tolerability of vortioxetine at higher than usual doses in complex clinical settings, even in a shorter time frame. This dosing strategy reflects clinical decisions made in a treatment-resistant population and is supported by vortioxetine’s favorable safety profile. Previous studies and real-world data suggest that upward titration may be feasible and well-tolerated, even beyond the approved range for depression, particularly when standard doses prove insufficient in severe or refractory cases. A recently published umbrella review by Wang et al. (29) indicates that the recommended range of vortioxetine doses for adults with MDD is generally 5–20 mg/day, with 20 mg/day emerging as the dose most consistently linked to robust improvements in depressive symptoms, cognition, anxiety, and quality of life (30, 31). Importantly, the review suggests that while 10 mg/day is often effective for depression, a clear dose-response pattern supports further benefit at 20 mg/day, without a concomitant, clinically prohibitive increase in side effects. However, the dose range reported by Wang et al. (29) may not fully account for the characteristics of OCD. Given that OCD often proves more resistant and may require more aggressive antidepressant dosing strategies, our use of vortioxetine up to 40 mg/day aligns with the broader principle that 20 mg/day is typically the upper standard dose for MDD and related conditions, yet individual patients with refractory features may benefit from a cautious exploration of higher doses. Our study’s retrospective design and real-world setting did not allow for the systematic evaluation of higher doses of vortioxetine in all patients. As a result, we were unable to draw firm conclusions about the relative benefits or risks of escalating vortioxetine beyond 20 mg/day in this population. Future prospective, dose-ranging research in SSRI-resistant OCD will be essential to clarify the potential added value of doses above the conventional day limit.

The tolerability profile of vortioxetine observed in our study aligns with findings from previous clinical trials and meta-analyses. In our sample, the most frequently reported adverse event was nausea, consistent with the RECONNECT and TRUE studies, which also identified it as the most common side effect. Similarly, the network meta-analysis by Kishi et al. (32) confirmed an increased incidence of nausea and vomiting associated with vortioxetine compared with placebo. However, no serious adverse events leading to discontinuation were observed in our study, in contrast to the RECONNECT study, in which two patients discontinued treatment due to dysgeusia and hypersensitivity, and the TRUE study, in which one serious adverse event (0.8%) was reported. Notably, the mean daily dose in our study (25.9 mg/day, with 10% of patients reaching 40 mg/day) was higher than the standard 10–20 mg/day used in the two other studies, but the tolerability remained comparable. The tolerability profile of vortioxetine contrasts with second-line strategies such as high-dose SSRI or augmentation with antipsychotics, which may carry a greater risk of side effects (3, 13). Further research is needed to clarify whether individual susceptibility to adverse effects of vortioxetine varies with dose and patient characteristics. Although SSRIs remain the first-line therapy (4), approximately 40–60% of patients do not attain complete remission (13), highlighting the need for alternative approaches. The combination of 5-HT1A agonism and 5-HT3/5-HT7 antagonism inherent in vortioxetine has been proposed to improve cognitive flexibility and reduce obsessive thoughts (19). Our data, showing significant changes in the Y-BOCS alongside tolerable side effects, suggest that this multimodal mechanism may translate into clinically meaningful benefits for at least a subset of patients with SSRI-resistant OCD. From a clinical perspective, vortioxetine may be considered as a monotherapy option in SSRI-resistant OCD, particularly in patients who have experienced poor tolerability or limited benefit from augmentation strategies. When using doses above 20 mg/day, we recommend close monitoring of tolerability and symptom progression during the first 2–4 weeks. Informed consent should include discussion of the off-label nature of this indication. In our sample, no serious adverse events were reported, and even higher doses were well tolerated, which supports its safety in real-world settings when appropriately monitored.

Despite these promising results, several limitations warrant caution. First, the lack of a control group precludes definitive conclusions about the efficacy of vortioxetine compared with other treatments. Second, the observational design and the relatively short follow-up period of 8 weeks limit the assessment of long-term outcomes and relapse rates. In addition, the study did not include functional or patient-reported outcome measures that could have provided further insight into the clinical relevance of symptom changes. Third, our sample size, although adequate for a preliminary assessment, limits the statistical power to detect small differences or to conduct meaningful subgroup analyses (e.g., by specific OCD symptom dimensions). Fourth, we did not systematically control for concomitant psychosocial interventions or for previous SSRIs, doses and treatment duration, which may have influenced response. Finally, the non-randomized selection of patients, based on clinician judgment for vortioxetine initiation, may have introduced bias that could limit the generalizability of our findings. In addition, the limited sample size may have reduced the statistical power to detect small or moderate effects and precluded subgroup analyses based on clinical or demographic variables. As a result, the findings should be interpreted as preliminary and exploratory. Given the retrospective design and absence of a control group, causal interpretations must be made with caution. Observed improvements could partially reflect factors unrelated to vortioxetine itself, including spontaneous symptom fluctuation, regression to the mean, or contextual therapeutic effects. Future randomized controlled trials will be necessary to isolate the drug’s specific contribution. Although we adopted a ≥25% Y-BOCS reduction as the threshold for treatment response, more stringent criteria such as a 35% reduction have been proposed (33), which may limit direct comparability with other recent studies. Moreover, we were unable to systematically retrieve data on the time elapsed between OCD onset and the initiation of first-line treatment, which may have influenced illness course and treatment responsiveness.

## 5 Conclusion

This retrospective study provides preliminary evidence supporting the potential efficacy and tolerability of vortioxetine in patients with SSRI-resistant OCD. The observed reduction in obsessive-compulsive, depressive, and anxiety symptoms highlights the potential of multimodal serotonergic modulation for refractory cases. Future studies should include randomized controlled trials comparing vortioxetine not only to placebo but also to established pharmacological strategies such as SSRI augmentation with antipsychotics. Trials should adopt standardized responder criteria (e.g., ≥35% Y-BOCS reduction) and incorporate multidimensional outcome measures, including functional status, quality of life, and cognitive flexibility. Stratification by prior treatment history and symptom dimensions may also help identify patient subgroups most likely to benefit from vortioxetine monotherapy. Longitudinal follow-up and multidimensional outcome measures would help clarify the durability and magnitude of therapeutic effects. If confirmed, vortioxetine could become part of a broader pharmacological toolkit aimed at improving outcomes in this challenging patient population.

## Data Availability

The raw data supporting the conclusions of this article will be made available by the authors, without undue reservation.
